# Effect of ST36 Acupuncture on Hyperventilation-Induced CO_**2**_ Reactivity of the Basilar and Middle Cerebral Arteries and Heart Rate Variability in Normal Subjects

**DOI:** 10.1155/2014/574986

**Published:** 2014-07-14

**Authors:** Sang-Ho Hyun, Jin-Wook Im, Woo-Sang Jung, Ki-Ho Cho, Young-Suk Kim, Chang-Nam Ko, Jung-Mi Park, Seong-Uk Park, Seung-Yeon Cho, Sang-Kwan Moon

**Affiliations:** Department of Cardiology and Neurology of Korean Medicine, College of Korean Medicine, Kyung Hee University, No. 26 Kyungheedae-ro, Dongdaemun-gu, Seoul 130-872, Republic of Korea

## Abstract

This study was conducted to verify the effect of acupuncture on cerebral haemodynamics to provide evidence for the use of acupuncture treatment as a complementary therapy for the high-risk stroke population. The effect of ST36 acupuncture treatment on the hyperventilation-induced CO_2_ reactivity of the basilar and middle cerebral arteries was studied in 10 healthy male volunteers (mean age, 25.2 ± 1.5 years) using a transcranial Doppler sonography with an interval of 1 week between measurements, and a portable ECG monitoring system was used to obtain ECG data simultaneously. The CO_2_ reactivity of the basilar and middle cerebral arteries increased significantly after ST36 acupuncture treatment, whereas the mean arterial blood pressure and pulse rate did not change significantly. The high-frequency power significantly increased after ST36 acupuncture treatment, and the percentage increase of high-frequency power correlated significantly with the percentage increase in the CO_2_ reactivity of the contralateral middle cerebral artery. These data suggest that ST36 acupuncture treatment increases CO_2_ reactivity, indicating improvement of vasodilatory potential of the cerebral vasculature to compensate for fluctuations caused by changes in external conditions. The increase in parasympathetic tone by ST36 acupuncture treatment is responsible for this therapeutic effect.

## 1. Introduction

Cerebral vessels respond to changes in the arterial carbon dioxide concentration in order to maintain constant cerebral blood flow (CBF), and this compensatory vasodilatory capacity of cerebral vessels is referred to as cerebral vasomotor reactivity (CVR). A decreased CVR is correlated with increasing age [1], cognitive impairment [2], white matter lesions [3], and stroke risks [4, 5]. Therefore, high-risk stroke populations must sustain a certain level of cerebral vasodilatory capacity. Recently, acupuncture has gained a reputation as an effective alternative treatment for cerebrovascular diseases because its efficacy for improving cerebral blood flow has been repeatedly reported [6–9]. These previous studies imply that acupuncture treatment can be used to improve CBF and CVR, thereby serving as an effective complementary therapy for recurrent stroke and other cerebrovascular diseases.

Previous studies have shown that sympathetic activation affects cerebral haemodynamics [10]. Increased sympathetic tone leads to constriction of the cerebral vessels in order to maintain a constant CBF under external stimulation, and this would be expected to reduce the dilative potential of the cerebral blood vessels during changes in carbon dioxide concentration. Zusanli (ST36), a distal acupoint that is located on the tibialis anterior muscle [11], was shown to lower sympathetic tone in previous studies [12]. Further, ST36 is repeatedly mentioned in old oriental medical documents for its use in preventing stroke [13], and its effect on cerebral blood flow has been reported in several studies [6, 8, 9]. These findings suggest that ST36 acupuncture treatment would have a beneficial effect on CVR via regulation of the autonomic nervous system. However, the effect of ST36 acupuncture on CVR has not been studied previously.

Although the effect of acupuncture treatment on CVR has been studied [14], the evidence remains scant. Only local acupoints near cerebral vessels were studied for their effectiveness with respect to CVR, and the mechanism of the effect of acupuncture on the cerebral vasculature has not been defined. Further, to our knowledge, the effect of acupuncture on the CVR of the posterior circulation has not been investigated. Because the CO_2_ reactivities of the anterior and posterior circulations show regional differences [15], each territory must be investigated individually. Therefore, our study was designed to provide data that are lacking from the previous studies. In the present study, we assessed the effect of the ST36 acupoint on hyperventilation-induced CO_2_ reactivity and corrected cerebral flow velocities (at P_ETCO_2__ = 40 mmHg) of both middle cerebral arteries (MCAs) and basilar artery (BA). Moreover, to establish a theory explaining the mechanism of the improvement in CVR, heart rate variability (HRV) data were collected simultaneously for 10 healthy young male volunteers.

## 2. Materials and Methods 

### 2.1. Subjects

This study included 10 healthy male volunteers (mean age, 25.2 ± 1.5 (S.D.) years; range, 23–27 years); all volunteers signed written consent forms. None of the subjects had a history of cerebrovascular disease, hypertension, diabetes mellitus, thyroid disease, or psychiatric disorders. Alcohol, coffee, smoking, or any other drugs were forbidden for 24 hours prior to examination. The Institutional Review Board of the Hospital of Korean Medicine, Kyung Hee Medical Center, approved the study protocol.

### 2.2. Intervention

All acupuncture procedures were performed by 1 experienced acupuncturist with a stainless steel acupuncture needle with a diameter of 0.25 mm and a length of 40 mm (Dong Bang Acupuncture, Seoul, Korea). The selected acupoint, ST36, is located on the tibialis anterior muscle 3-cun inferior to ST35, which is located on the anterior aspect of the knee, in the depression lateral to the patellar ligament [11]. The needle was inserted into the skin to a depth of approximately 15 mm at left ST36 acupoint, and stronger stimulation was applied by physical rotation of the needle for 10 seconds at 2 Hz immediately after insertion. The needle was removed after 20 minutes.

### 2.3. Measurement

The cerebral blood flow velocities and the CO_2_ reactivities of both MCAs and the BA were measured by transcranial Doppler sonography (TCD) using a Multi-Dop X4 system (Compumedics DWL, Singen, Germany), as described in previous studies [14, 16, 17]. On the first visit, the volunteer maintained a supine position, and the CBF velocities of both MCAs were measured through both temporal windows. On the second visit, 1 week later, the volunteer remained in a seated position, and the CBF velocity of the BA was measured through the suboccipital window by using a 2-MHz pulsed-Doppler probe. We attached a removable bilateral probe holder (LAM-Rack; Compumedics DWL) for both temporal windows and a probe-holding device for the suboccipital window to avoid shifting of the probe and to allow continuous measurement. The strongest wave signal was sought at depths ranging from 45 to 60 mm for the MCAs and from 75 to 110 mm for the BA, and the sample and gain values were adjusted and were recorded when the waveform remained constant. All measurements commenced after the subjects had stabilized for 5 minutes. The mean blood flow velocity was calculated continuously as the time-averaged maximum velocity over the cardiac cycle, as computed from the envelope of the maximum frequencies. The subject went through a 4-minute rest and a 1-minute hyperventilation period. The mean MCA and BA blood flow velocities were obtained at rest in the stable normocapnic condition and were obtained during the hyperventilation period under the hypocapnic condition. All TCD spectra were recorded for later review.

The blood flow velocity is dependent on the arterial CO_2_ tension; thus, the corrected blood flow velocity was calculated at 40 mmHg of CO_2_ tension (CV40, cm/s), as described previously [18]:

(1) CV40(Corrected  Velocity  at  PETCO2  40 mmHg) =V1·eb(PCO2  40 mmHg-P1CO2),

where *b* is the CO_2_ reactivity, *V*_1_ is the velocity at P_1_CO_2_, and P_ETCO_2__ is the end-tidal CO_2_ partial pressure.

CO_2_ reactivity refers to the percent change in mean blood velocity per change in P_ETCO_2__, as calculated by the following formula [17]:

(2) $$\begin{array}{c} {\text{CO}}_{2}\;\;\text{reactivity}=100\times \frac{\left[V_{\text{rest}}-V_{\text{hypocapnia}}\right]/V_{\text{rest}}}{\Delta {\text{P}}_{{\text{ETCO}}_{2}}}, \end{array}$$

where *V*_rest_ is the blood flow velocity at rest, obtained during the most stable 10 seconds in the stable normocapnic condition, *V*_hypocapnia_ is the blood flow velocity obtained during the most stable 10 seconds of the latter half of the 1-minute hyperventilation period, and ΔP_ETCO_2__ is the change in P_ETCO_2__ from baseline to maximal hyperventilation. The CO_2_ reactivity was expressed as %/min.

**Figure 1:**
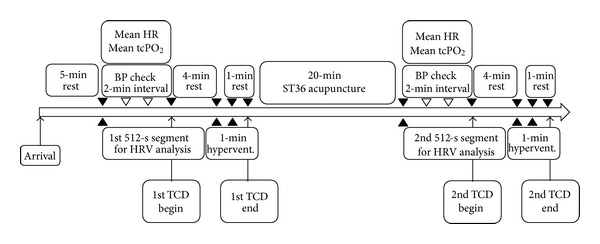
Timeline of the study design. HR, heart rate; tc, transcutaneous; BP, blood pressure; HRV, heart rate variability; TCD, transcranial Doppler sonography; hypervent., hyperventilation.

The variables that may control CBF, such as blood pressure, heart rate, P_ETCO_2__, and transcutaneous (tc) PO_2_, were observed by using various modules of the Cardiocap S/5 collector (Datex-Ohmeda, Helsinki, Finland). Blood pressure and heart rate were measured under normocapnic conditions before hyperventilation. Blood pressure was measured 4 times at 2-minute intervals to determine the mean blood pressure. Heart rate and tcPO_2_ were continuously monitored by an oximetry apparatus positioned on the subject's finger. A Cardiocap S/5 collector-connected nasal prong positioned in front of the subject's nostril monitored P_ETCO_2__, and each subject was instructed to breathe only through the nose during the study. A snapshot function in the Cardiocap S/5 collector software program was used to calculate the mean heart rate, mean tcPO_2_, and P_ETCO_2__ at specific time points during the study. These 4 variables were monitored and the data were saved in the computer, which was connected to the Cardiocap S/5 collector. The measurements were performed before and after the 20-minute ST36 acupuncture treatment (Figure 1).

ECG data were obtained simultaneously by using an ECG monitoring system (FM-150; Fukuda Denshi, Japan), with a sampling rate of 125 Hz. ECG monitoring was continued throughout the procedure, and the obtained ECG data were transferred to a PC from the IC card of the recorder. Power spectral analysis of the RR-interval variability was performed by 512-point fast Fourier transformation using SCM-510 (Fukuda denshi, Japan), a HRV analysis software program, to obtain the following HRV parameters: low-frequency power (LF power, ms^2^), high-frequency power (HF power, ms^2^), and the LF/HF ratio in the frequency domain [19]. Segments of 512-second duration before and after the ST36 acupuncture treatment were used for the analysis (Figure 1).

### 2.4. Data Analysis

Statistical analysis was performed by using the Statistical Package for the Social Sciences version 12.0 for Windows (SPSS, Chicago, IL). The data are summarized as the means ± standard deviation. The Wilcoxon signed-rank test was used for statistical comparisons between the values before and after ST36 acupuncture treatment. We correlated the changes in CO_2_ reactivity with both changes in the CV40 and the HRV parameters, by using the Pearson correlation. *P* values < 0.05 were considered significant.

## 3. Results

**Figure 2:**
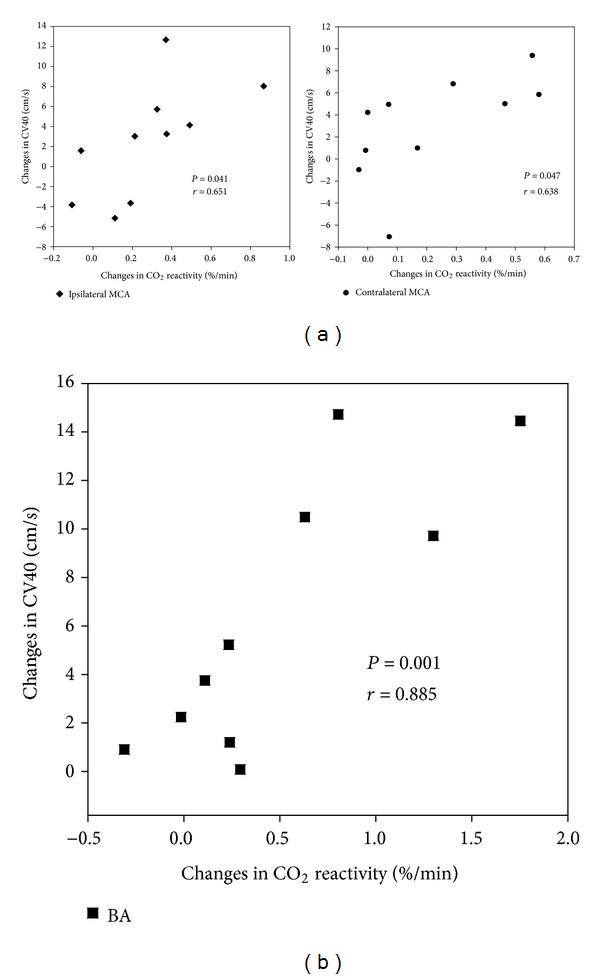
Correlation analysis between the changes in CO_2_ reactivity and the changes in CV40 before and after ST36 acupuncture. (a) A significant correlation is shown between the changes in CO_2_ reactivity and the CV40 of the ipsilateral MCA (*P* = 0.041, *r* = 0.651) and the contralateral MCA (*P* = 0.047, *r* = 0.638). (b) A significant correlation is shown between acupuncture-mediated changes in CO_2_ reactivity and the CV40 of the BA (*P* = 0.001, *r* = 0.885).

No side effects occurred during acupuncture treatment. The mean blood pressure and heart rate showed no significant changes after ST36 acupuncture treatment (*P* = 0.721 and *P* = 0.386, resp.). Significant increases were observed in the CO_2_ reactivities of both MCAs and the BA during hypocapnia (Table 1) and in the corrected blood flow velocity (at P_ETCO_2__ = 40 mmHg) of the BA, whereas the CV40 of both MCAs showed a nonsignificant increasing trend after ST36 acupuncture treatment (Table 2). A positive linear correlation was observed between the acupuncture-mediated changes in CO_2_ reactivity and the CV40 of both MCAs and the BA (Figure 2).

**Figure 3:**
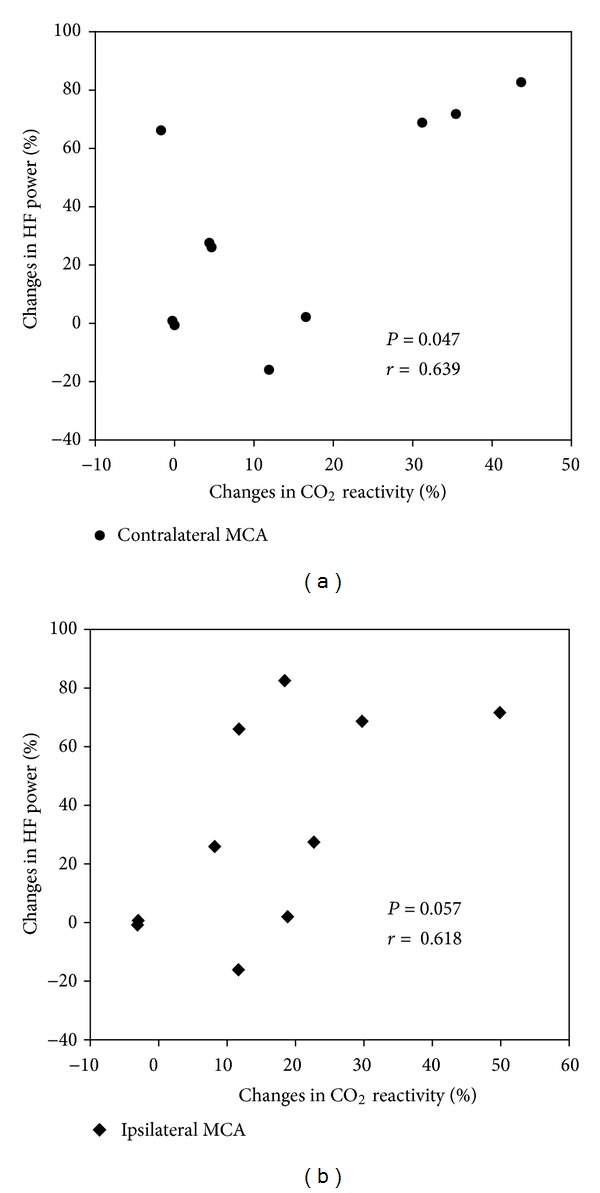
Correlation analysis between the percentage changes in CO_2_ reactivity and HF power before and after ST36 acupuncture treatment. (a) A significant correlation is shown between acupuncture-induced changes in HF power and the CO_2_ reactivity of the contralateral MCA (*P* = 0.047, *r* = 0.639). (b) The correlation between the percentage changes in the CO_2_ reactivity of the ipsilateral MCA and HF power was slightly greater than the significance cut-off (*P* = 0.057, *r* = 0.618).

The HF power increased significantly and the LF/HF ratio decreased significantly after ST36 acupuncture treatment (Table 3). The percentage changes in the HF power were significantly correlated with the percentage changes in CO_2_ reactivity induced by ST36 acupuncture treatment in the contralateral MCA (Figure 3).

## 4. Discussion

The purpose of the present study was to verify the effect of ST36 on blood flow velocity and the CO_2_ reactivity of both MCAs and the BA during hypocapnia in normal subjects and to determine whether the autonomic nervous system plays a role in the improvement of cerebral haemodynamics. Hyperventilation-induced CO_2_ reactivity of both MCAs and the BA increased significantly after application of ST36 acupuncture treatment, while the mean blood pressure and pulse rate did not change significantly. Because patients with stroke risk factors and stroke survivors show impaired CO_2_ reactivity [4, 20] and because reduced CVR implies a greater risk of ipsilateral stroke and transient ischemic attack [5], the results of this study suggest that ST36 acupuncture treatment can be used as a nonpharmacological intervention to improve the cerebral haemodynamics of the anterior and posterior circulation. Along with CO_2_ reactivity, an ambulatory ECG monitoring system was used to obtain HRV data simultaneously, in order to determine whether acupuncture-induced activation of sympathetic or parasympathetic tone plays a role in increasing CVR. A significant increase in the HF power and a significant decrease in the LF/HF ratio were observed, which implies increased activation of parasympathetic tone [21]. In the correlation study, increases in the HF power were significantly correlated with increases in the CO_2_ reactivity of the contralateral MCA (*P* = 0.047), but the correlation with increases in the CO_2_ reactivity of the ipsilateral MCA was slightly greater than the significance cut-off (*P* = 0.057). This suggests that acupuncture-induced activation of parasympathetic tone plays a role in the improvement of CO_2_ reactivity.

A significant increase in the mean corrected blood flow velocity (at P_ETCO_2__ = 40 mmHg) of the BA was observed after ST36 acupuncture treatment. We measured all of the variables that can influence CBF and blood flow velocities: mean blood pressure, heart rate, PCO_2_, and arterial oxygen content [22, 23]. The tcPO_2_, mean blood pressure, and heart rate did not change significantly after ST36 acupuncture treatment. We assessed P_ETCO_2__ instead of PCO_2_ because if P_ETCO_2__ is not influenced by changes in arterial blood pressure, it represents a close approximation of the arterial PaCO_2_ [24]. The percentage change in CV40 may be proportional to the percentage change in the CBF of the corresponding territory if the vessel diameter is constant because the P_ETCO_2__ response curves for the blood flow velocity of the MCA resemble those of the PCO_2_ response curves for CBF [18], and the blood flow velocity in defined cerebral arteries is significantly positively correlated with the corresponding regional CBF [25]. Cerebral arteries larger than 0.57 mm in diameter appear to act only as conductance vessels because significant changes in the diameters of cerebral arteries were not observed under changes in PCO_2_ in humans [26], and the diameters of the MCA and the BA vary from 2.5 to 4 mm (mean, 3.35 mm) and from 3 to 7 mm (mean, 4.3 mm), respectively [27, 28]. Thus, the acupuncture-induced increase in the CV40 of the BA indicates that the CBF of the BA territory had increased, and this rheological improvement of cerebral vasculature resulted in an improvement of the CVR. On the other hand, the CV40 increase of both MCAs showed a nonsignificant tendency to increase; therefore, this result should be interpreted with caution. Cerebrovascular CO_2_ reactivity is known to be directly related to the regional CBF and inversely related to blood pressure [29]. Because the mean blood pressure did not change significantly after acupuncture treatment in this study, the acupuncture-induced increase in the CO_2_ reactivity of both MCAs suggests that the corresponding regional CBF had increased. Further, significant correlations between the increases in CV40 and CO_2_ reactivity of both MCAs and the BA were observed in this study, indicating that the CO_2_ reactivity shows a good correlation with both the CV40 and the regional CBF. Although the acupuncture-induced changes in the CV40 of the ipsilateral and contralateral MCAs only showed nonsignificant increasing trend (*P* = 0.241 and *P* = 0.114, resp.), ST36 acupuncture treatment significantly increased CO_2_ reactivity of both MCAs, suggesting that the CBF had increased in both MCA territories. Further studies that are performed under similar conditions are needed to confirm the present findings.

Numerous previous studies [6, 8, 9] support the effect of ST36 acupuncture treatment on cerebral haemodynamics. We previously used single photon emission computed tomography (SPECT) to demonstrate that electroacupuncture at right ST36 and ST41 acupoints significantly increased the regional CBF in both inferior parietal lobes [9]. Further, Litscher et al. [8] showed that acupuncture treatment at ST36, PC6, SP6, and CV6 significantly increased the mean blood flow velocity of the right MCA by using TCD. According to Song et al. [6], who used SPECT to investigate the effect of the ST36 acupoint, acupuncture at right ST36 increased the regional CBF in the left anterior temporal lobe, right inferior frontal lobe, and left cerebellum. Further, Cho et al. [30] used functional magnetic resonance imaging (fMRI) to show that acupuncture stimulation at left ST36 displayed significantly higher activation in the left frontoparietal lobe, right occipital lobe, right cerebellar lobe, and right pons, compared to pressure stimulation at left ST36. These previous studies indicate that ST36 increases the CBF in both MCA territories and the BA territory, which is consistent with the results of the present study.

ST36 acupuncture treatment increased CO_2_ reactivity in 20 minutes in the present study, and it is unlikely that major changes in arterial geometry were responsible for such an immediate response. Autonomic nervous system activation and improvement of endothelial function are possible explanations for this rapid acupuncture-mediated increase in CVR [12, 31]. A previous study reported the endothelial function improving effect of ST36 acupuncture treatment [31], and other studies suggested that ST36 acupuncture treatment increases and activates endothelial NO synthesis [32]. Endothelial function is impaired in patients with lacunar infarction, small vessel disease, and atherosclerosis [33, 34], and these patients exhibit reduced nitric oxide-mediated vessel relaxation and vascular tone abnormality [35]. Based on these previous findings, it is likely that ST36 acupuncture treatment increased the CO_2_ reactivity of both MCAs and the BA by improving endothelial function, but CO_2_ reactivity and endothelial function must be assessed simultaneously before and after acupuncture application in a future study in order to confirm this mechanism.

A limitation of the present study is the lack of a control group; however, we have obtained TCD-measured CVR data in our previous studies that can serve as a historical control group. The CO_2_ reactivity of both MCAs in 10 healthy young male volunteers (age, 26.1 ± 1.8 years) who took placebo drugs was measured by using a protocol similar to that of the current study, and no significant change was observed [36]. In our previous study (unpublished data), we found that acupuncture treatment at GB20 produced no significant change in the CO_2_ reactivity of the ipsilateral MCA (*P* = 0.496) or the contralateral MCA (*P* = 0.191) in 15 healthy young male volunteers (age, 25.6 ± 1.8 years) when using methods and devices identical to those used in the present study to investigate CO_2_ reactivity. Therefore, although this study did not have a control group, data from our previous studies support the efficacy of ST36 acupuncture treatment on improvement of CO_2_ reactivity in both MCAs. Another limitation of our study is the small sample size and incomplete demonstration of the mechanism of acupuncture-induced improvement of CO_2_ reactivity. While the percentage changes in the HF power correlated with the percentage changes in CO_2_ reactivity in the contralateral MCA, the correlation between the percentage changes in HF power and CO_2_ reactivity of the ipsilateral MCA was not significant. A great number of subjects should be studied to confirm this theory. Further, other mechanisms such as improvement of endothelial function should be studied in the future. Our results should encourage further studies regarding the effectiveness of acupuncture treatment on improving cerebral haemodynamics in the high-risk stroke population.

## 5. Conclusion

ST36 acupuncture treatment increased the CO_2_ reactivity of both MCAs and the BA during hypocapnia in healthy subjects, which indicates increased vasodilatory potential of the cerebral vasculature to compensate for fluctuations caused by changes in external conditions. Further, the application of acupuncture treatment on ST36 increased the blood flow velocity of the BA, suggesting that the CBF in the BA territory had been increased. Changes in HRV parameters represent an increase in parasympathetic tone due to ST36 acupuncture, and increases in the CO_2_ reactivity of the contralateral MCA were significantly correlated with increases in the HF power, showing that autonomic nervous system activation plays a role in acupuncture-mediated improvement of cerebral haemodynamics.

## Figures and Tables

**Table 1:** Changes in hyperventilation-induced CO_2_ reactivity (%/min) before and after ST36 acupuncture.

	Before	After	*P* value
Ipsilateral MCA	1.92 ± 0.62	2.19 ± 0.56	0.013
Contralateral MCA	1.71 ± 0.41	1.92 ± 0.35	0.022
BA	2.78 ± 0.76	3.29 ± 0.85	0.037

MCA, middle cerebral artery; BA, basilar artery.

**Table 2:** Changes in corrected blood flow velocity at P_ETCO_2__ = 40 mmHg (CV40, cm/sec) before and after ST36 acupuncture.

	Before	After	*P* value
Ipsilateral MCA	58.1 ± 11.7	60.7 ± 14.4	0.241
Contralateral MCA	58.3 ± 11.0	61.2 ± 11.7	0.114
BA	29.2 ± 6.6	35.5 ± 10.6	0.005

MCA, middle cerebral artery; BA, basilar artery.

**Table 3:** Changes in HRV parameters before and after ST36 acupuncture.

	Before	After	*P* value
LF power (ms^2^)	1437.1 ± 805.9	1280.4 ± 820.1	0.721
HF power (ms^2^)	856.2 ± 464.6	1072.1 ± 457.4	0.037
LF/HF ratio	1.87 ± 1.04	1.26 ± 0.71	0.028

HRV, heart rate variability; LF, low-frequency; HF, high-frequency.
